# Why Cemiplimab? Defining a Unique Therapeutic Niche in First-Line Non-Small-Cell Lung Cancer with Ultra-High PD-L1 Expression and Squamous Histology

**DOI:** 10.3390/cancers18020272

**Published:** 2026-01-15

**Authors:** Satoshi Ikeda, Keigo Araki, Mai Kitagawa, Natsuno Makihara, Yutaro Nagata, Kazuki Fujii, Kiyori Yoshida, Tatsuki Ikoma, Kahori Nakahama, Yuki Takeyasu, Utae Katsushima, Yuta Yamanaka, Takayasu Kurata

**Affiliations:** Department of Thoracic Oncology, Kansai Medical University, 2-5-1 Shin-machi, Hirakata 573-1010, Japankurata.tak@kmu.ac.jp (T.K.)

**Keywords:** non-small cell lung cancer, squamous cell carcinoma, cemiplimab

## Abstract

The landscape of first-line treatment for metastatic non-small cell lung cancer (NSCLC) without actionable driver mutations is rapidly evolving, currently dominated by pembrolizumab-based regimens. This review discusses the unique molecular characteristics of cemiplimab, a newer anti-PD-1 antibody, and defines its optimal positioning against established standards. Cemiplimab is a fully human IgG4 monoclonal antibody distinguished by two key features: an engineered hinge-region mutation that prevents Fab-arm exchange, ensuring exceptional molecular stability which minimizes anti-drug antibody (ADA) risks associated with unstable molecules; and a unique interaction with PD-1 glycosylation sites, potentially enhancing binding efficacy. These structural advantages may be particularly relevant in histologies like squamous NSCLC, where accumulating somatic mutations drive high neoantigen loads and heightened immune responses, creating an environment historically prone to ADA formation. Based on data from the pivotal EMPOWER-Lung program, we highlight cemiplimab’s exceptional promise in specific populations. Firstly, in the EMPOWER-Lung 1 trial, cemiplimab monotherapy demonstrated extraordinary survival benefits in a pre-specified analysis of the distinct “ultra-high” PD-L1 expression subgroup (TPS ≥90%), potentially surpassing historical benchmarks. Secondly, cemiplimab displays consistent, robust efficacy in challenging-to-treat squamous histology, both as monotherapy for patients with high PD-L1 expression and in combination with chemotherapy for patients with PD-L1 < 50%. In conclusion, cemiplimab establishes a unique therapeutic niche for patients with squamous histology and ultra-high PD-L1 expression, likely driven by its distinct structural stability and reduced immunogenicity.

## Simple Summary

This review evaluates the role of cemiplimab, an anti-PD-1 antibody, analyzing data from the pivotal EMPOWER-Lung1 and EMPOWER-Lung3 trials. The findings highlight cemiplimab’s robust efficacy across difficult-to-treat subgroups, including those with squamous histology and brain metastases. A key distinction is its exceptional performance in patients with “ultra-high” PD-L1 expression (≥90%). We discuss the plausible biological mechanism linking cemiplimab’s unique structural stability to reduced anti-drug antibody risks, potentially enhancing efficacy in these highly immunogenic tumors. Additionally, the combination of cemiplimab and chemotherapy offers a strong alternative for lower PD-L1 expression levels. In conclusion, cemiplimab represents a critical therapeutic option, potentially establishing a unique niche for specific, high-need Non-Small Cell Lung Cancer populations.

## 1. Introduction

Lung cancer remains the leading cause of cancer-related mortality and presents a persistent challenge to the oncology community [1]. For many decades, treatment options were quite limited for patients with metastatic non-small cell lung cancer (NSCLC) who did not have specific genetic mutations—such as *EGFR* and *ALK*—that could be targeted with drugs [2]. During this time, the standard treatment was almost exclusively platinum-based cytotoxic chemotherapy [3]. While chemotherapy was able to shrink tumors for a short time, these treatments had significant drawbacks. Patients often suffered from severe side effects, and the positive results were usually temporary [4]. Consequently, there was a desperate need for new treatments that could keep the disease under control for a longer period.

Everything changed with the arrival of immune checkpoint inhibitors (ICIs). Specifically, drugs that target the Programmed Cell Death-1 (PD-1) receptor or its partner, PD-L1, have completely revolutionized how we treat this disease [5]. Instead of attacking cancer cells directly like chemotherapy, these new drugs work by blocking the “brakes” that stop the immune system from working [6]. By removing these barriers, they wake up the patient’s own T cells, allowing the immune system to recognize and destroy cancer cells. This approach has offered the possibility of long-term survival, something that was previously impossible to achieve [7].

The first wave of these PD-1 inhibitors, most notably pembrolizumab and nivolumab, established this type of therapy as a foundation of modern cancer care [8,9]. However, the number of approved drugs has grown rapidly, adding a new layer of complexity for doctors. Clinicians now face a difficult task: they must choose the best option from a growing list of drugs that, on the surface, seem to target the same biological pathway. However, these drugs are not all the same. Small differences in how the antibodies are built—such as their structure, how they were engineered, where exactly they bind to the target, and how they interact with sugar molecules (glycosylation)—can lead to meaningful differences in how well they work, how safe they are, and how the body reacts to them [10,11].

Cemiplimab (Libtayo^®^) represents a sophisticated step forward in this class of medications. It is a fully human monoclonal antibody with high affinity, designed with a specific structure known as IgG4 [12]. While approval status varies by country, cemiplimab has been approved for cutaneous squamous cell carcinoma [13], basal cell carcinoma [14], and cervical cancer [15]. Regarding NSCLC, it was approved by the United States in February 2021 and by Europe in June 2021. Subsequently, it was approved in Japan in September 2025. This approval covers a wide range of uses: it can be used as monotherapy for patients with high PD-L1 levels, or in combination with chemotherapy for a broader group of patients.

The purpose of this review is to answer the critical question: “Why choose Cemiplimab?” To do this, we will examine its unique structural biology in detail. We will specifically look at its stabilized backbone and its unique binding method that depends on the N58-glycan, comparing these special features with other well-known inhibitors. Furthermore, we will analyze important long-term data from the EMPOWER-Lung clinical trials. Based on this evidence, we propose that cemiplimab fills a unique and potentially superior role in therapy, particularly for patients with “ultra-high” PD-L1 expression and those with squamous NSCLC.

## 2. Molecular Characteristics

### 2.1. The IgG4 Backbone and the S228P Mutation: Ensuring Stability and Low Immunogenicity

To truly understand why cemiplimab is unique in a clinical setting, we must look closely at how the molecule was designed. Monoclonal antibodies are not simple drugs; they are complex proteins known as glycoproteins. Even tiny changes in their shape or amino acid sequence can have a huge impact on how long they last in the bloodstream and how well they attach to their targets. Therefore, the specific engineering choices made for cemiplimab give us a strong reason to believe it works differently than older drugs.

Cemiplimab is built using a specific type of antibody framework called Immunoglobulin G4 (IgG4). In the world of cancer immunotherapy, scientists often choose IgG4 instead of the more common IgG1. The reason for this choice is safety. IgG4 has a lower tendency to interact with other parts of the immune system (specifically, it has low affinity for Fc gamma receptors and C1q) than IgG1 [16].

However, naturally occurring human IgG4 antibodies possess a unique structural instability known as “Fab-arm exchange” [16]. In vivo, the heavy chains of IgG4 molecules can dissociate and re-associate with heavy chains from other disparate IgG4 molecules present in the plasma. This phenomenon results in the formation of bispecific antibodies that are functionally monovalent for their original target. Such structural fluidity can lead to unpredictable pharmacokinetics and reduced therapeutic efficacy, as the antibody loses the avidity gained from bivalent binding [17].

**Figure 1:**
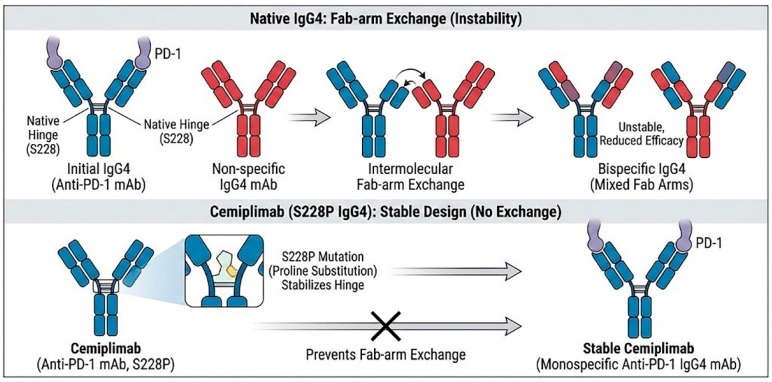
Prevention of Fab-arm exchange by the S228P mutation in the IgG4 hinge region of Cemiplimab. The S228P mutation stabilizes the hinge, preventing the formation of bispecific antibodies and ensuring a stable, monospecific therapeutic molecule.

To fix this instability, cemiplimab uses a specific design change: a serine-to-proline substitution at amino acid position 228 (S228P) in the hinge region (Figure 1). This mutation introduces rigidity to the inter-chain disulfide bonds, effectively mimicking the stable hinge structure of an IgG1 antibody while retaining the low effector function of IgG4 [18]. This engineering ensures that cemiplimab remains a stable, bivalent molecule capable of high-affinity binding throughout its circulation time.

### 2.2. Anti-Drug Antibodies (ADAs) and Immunogenicity

Therapeutic monoclonal antibodies can be recognized as foreign antigens by the patient’s immune system, potentially inducing the production of anti-drug antibodies (ADAs). The formation of ADAs is a critical factor in treatment failure, as they can accelerate the clearance of the drug from the body, leading to reduced blood concentrations, and may also directly neutralize the drug’s biological activity, thereby attenuating its efficacy [19]. In fact, the correlation between ADA production and reduced efficacy was demonstrated in the clinical trials evaluating atezolizumab. A pooled analysis revealed that patients who developed treatment-emergent ADAs exhibited a less favorable hazard ratio for overall survival (HR 0.89) compared to those who remained ADA-negative (HR 0.68), suggesting that the accelerated drug clearance associated with ADAs can attenuate the therapeutic benefit [20,21].

In the context of ICIs, data indicate that cemiplimab may possess a favorable immunogenicity profile, with a lower incidence of ADA production compared to other existing ICIs. Clinical data indicate a remarkably low incidence of treatment-emergent ADAs, ranging from approximately 0% to 2.6% [22,23]. This suggests that the stabilized IgG4 structure of cemiplimab is viewed as “self” by the human immune system and is highly stable in vivo. In marked contrast, a systematic review comprising 141 trials across 16 tumor types revealed considerably higher immunogenicity profiles for other widely used checkpoint inhibitors [24]. Specifically, atezolizumab demonstrated the highest incidence of treatment-emergent ADAs, with rates reported at 29.6% in large-scale studies and ranging up to 54.1% across individual trials [21,24,25]. Nivolumab also exhibits a comparatively high immunogenicity profile, with pooled FDA data indicating an ADA incidence of 11.2% [26]. While pembrolizumab generally displays a lower immunogenicity profile comparable to cemiplimab in pooled analyses, incidences as high as 20% have been documented in certain trial settings. Collectively, these comparative data underscore the unique stability of cemiplimab’s molecular design in minimizing immunogenic responses relative to other agents in the class.

Generally, the production of ADAs is more likely to occur in physiological states characterized by heightened immune function. Squamous NSCLC is typically associated with a high tumor mutation burden (TMB) [27], where the accumulation of somatic mutations leads to the generation of neoantigens and a subsequent robust induction of immune responses [28]. Therefore, squamous NSCLC, characterized by heightened immune reactivity, inherently possesses a predisposition for ADA generation. In this context, prioritizing cemiplimab, which may exhibit a lower immunogenic potential compared with other ICIs, could offer a strategic advantage, potentially translating into durable responses and long-term survival benefits. Squamous tumors naturally trigger a strong immune response. However, the stability of the cemiplimab molecule appears to be the main factor preventing the creation of ADAs.

### 2.3. Critical Role of PD-1 Glycosylation (N58-Glycan)

Recent mechanistic insights have elucidated that post-translational modifications of PD-1 play a critical role in immune checkpoint regulation. Specifically, glycosylation at the asparagine 58 (N58) position of PD-1 significantly enhances the affinity of the PD-1/PD-L1 interaction, thereby reinforcing T-cell suppression [29]. In this context, cemiplimab distinguishes itself from other anti-PD-1 agents, such as pembrolizumab and nivolumab, through its unique binding properties. Cemiplimab specifically recognizes and binds to the N58-glycosylated form of PD-1, a feature that may allow for more efficient steric inhibition of the PD-1/PD-L1 axis in tumors where this glycosylation is prevalent [30].

This structural differentiation may underlie the divergent clinical outcomes observed in squamous cell carcinomas. The glycosyltransferase B3GNT2 has been identified as a key enzyme driving PD-1 glycosylation and serving as a resistance factor to conventional immunotherapy; its expression levels inversely correlate with the efficacy of existing anti-PD-1 antibodies. Crucially, genomic analyses have revealed increased copy numbers of B3GNT2 specifically in lung squamous cell carcinoma [31]. In fact, in the adjuvant setting for cutaneous squamous cell carcinoma, a pivotal trial evaluating cemiplimab met its primary endpoint, whereas a comparable study involving pembrolizumab failed to demonstrate a similar benefit [32].

In tumors like squamous cell carcinoma, high PD-1 glycosylation strengthens the bond between PD-1 and PD-L1. This strong bond may block standard inhibitors from working. Cemiplimab, however, specifically targets these glycosylated variants. While this hypothesis requires validation in head-to-head clinical studies, cemiplimab may fill a critical therapeutic gap, offering potential efficacy in patient populations that are intrinsically resistant to other agents.

## 3. Evidence from Pivotal Trials

### 3.1. EMPOWER-Lung 1

The clinical utility of cemiplimab in NSCLC has been established through the robust EMPOWER-Lung program. These large Phase 3 trials were designed to test the drug in populations that reflect real-world clinical practice, providing the evidentiary basis for its specific therapeutic positioning.

The EMPOWER-Lung 1 study was a global Phase 3 trial. It compared cemiplimab alone versus standard platinum chemotherapy chosen by the investigator [33,34,35]. Patients could join if they had PD-L1 levels of 50% or higher. Those with EGFR, ALK, or ROS1 mutations were excluded. This study was designed with overall survival (OS) and progression-free survival (PFS) as the primary endpoints, assessed by a blinded independent central review (BICR). Key secondary endpoints included the objective response rate (ORR) and duration of response (DOR) per BICR, alongside safety evaluations. Additionally, exploratory analyses were pre-specified to evaluate efficacy outcomes stratified by PD-L1 expression levels (≥90%, 61–89%, and ≤60%).

During the enrollment phase, a significant challenge emerged regarding the accuracy of PD-L1 expression assessment. It was identified that PD-L1 testing performed at a specific laboratory facility did not meet quality standards, which affected a subset of the enrolled population. Consequently, re-testing of samples was required to verify eligibility. To address this issue and ensure the robustness of the data, the study defined a modified intention-to-treat (mITT) population. The mITT-1 population (*n* = 563) comprised patients with confirmed PD-L1 expression of ≥50%, including those randomized with valid initial testing (mITT-2, *n* = 475) and those from the affected group whose eligibility (PD-L1 ≥ 50%) was subsequently confirmed upon re-testing (*n* = 88). In agreement with regulatory authorities, sensitivity analyses for the primary endpoints were conducted within this mITT-1 population in addition to the primary analysis of the ITT population.

A notable feature of this trial was its ethical crossover design; patients who started on chemotherapy were allowed to switch to cemiplimab if their disease progressed. Despite a high effective crossover rate of 73.9%, cemiplimab demonstrated a statistically significant and clinically meaningful improvement in survival. In the 5-year update presented in 2024, the median OS for patients in the cemiplimab arm was 26.1 months (95% CI: 22.1–31.9), compared to 13.3 months (95% CI: 10.5–16.2) for the chemotherapy arm. This survival advantage was statistically significant, with a hazard ratio (HR) of 0.59 (95% CI: 0.48–0.72; *p* < 0.0001) in the population with confirmed PD-L1 ≥ 50%, with results favoring cemiplimab across generally all subgroups. The long-term durability of the response was further evidenced by the 5-year survival rate, which was nearly double for the immunotherapy group at 29.0%, versus 15.0% for those treated with chemotherapy. PFS also favored cemiplimab, with a median PFS of 8.1 months versus 5.3 months for chemotherapy (HR 0.50; 95% CI: 0.41–0.61; *p* < 0.0001). Consistent benefits favoring cemiplimab were observed across all subgroups.

The most striking finding from EMPOWER-Lung 1 came from a pre-specified analysis of patients with “ultra-high” PD-L1 expression, defined as a Tumor Proportion Score (TPS) of 90% or greater. In the subgroup of patients with PD-L1 ≥ 90%), cemiplimab demonstrated a substantial survival benefit compared with chemotherapy. The median OS was 38.8 months (95% CI: 22.9–NE) in the cemiplimab arm versus 13.7 months (95% CI: 8.8–20.6) in the chemotherapy arm, corresponding to a HR of 0.442 (95% CI: 0.303–0.645). Similarly, PFS was significantly prolonged with cemiplimab, with a median PFS of 14.7 months compared to 5.1 months for chemotherapy (HR 0.321; 95% CI: 0.222–0.464). Furthermore, the ORR was markedly higher in the cemiplimab group at 60.6% (95% CI: 50.3–70.3) versus 17.9% (95% CI: 10.8–27.1) in the chemotherapy group (odds ratio 7.080; 95% CI: 3.650–13.731). These findings underscore the particularly high expectations for cemiplimab as a therapeutic option for this specific population.

With a 5-year follow-up, the safety profile of cemiplimab remains manageable and consistent with the established profile of other anti–PD-1 monotherapies. Any-grade treatment-emergent adverse events (TEAEs) were reported in 62.9% of patients in the cemiplimab group, compared with 90.4% in the chemotherapy group. Notably, the incidence of Grade ≥ 3 TEAEs was lower with cemiplimab (18.3%) than with chemotherapy (39.9%). Treatment discontinuation due to TEAEs occurred in 7.3% of patients receiving cemiplimab. Overall, these data indicate no new safety signals or specific concerns relative to comparable regimens.

### 3.2. EMPOWER-Lung 3

While monotherapy is excellent for high expressors, many patients require the immediate tumor-shrinking power of chemotherapy combined with immunotherapy. The EMPOWER-Lung 3 trial addressed this need. This randomized, double-blind Phase 3 study tested the combination therapy. Patients received either cemiplimab (350 mg every 3 weeks) or a placebo, along with four cycles of platinum-based chemotherapy [36,37]. The trial enrolled 466 patients regardless of PD-L1 status or histology. The primary endpoint was OS. Key secondary endpoints included PFS and ORR, both of which were assessed by a blinded independent central review. Additionally, the trial evaluated DOR per central review, best overall response determined by either central or investigator assessment, and safety profiles.

The 5-year follow-up results released in late 2025 confirmed the long-term durability of this regimen [38]. In the overall population, the median OS was 21.1 months (95% CI: 15.9–23.9) with the cemiplimab plus chemotherapy versus 12.9 months (95% CI: 10.6–16.1) with chemotherapy alone (HR 0.66; 95% CI: 0.53–0.83). The 5-year survival rate was 19.4% for the cemiplimab plus chemotherapy arm compared to 8.8% for the chemotherapy arm. Consistent survival benefits favoring the cemiplimab plus chemotherapy regimen were generally observed across the majority of prespecified subgroups, including patients with squamous or non-squamous histology, those with a smoking history, and patients with PD-L1 expression of 1–49% (HR 0.50) or ≥50% (HR 0.56). While the overall trend was positive, the survival advantage appeared attenuated in the specific subgroups of patients with PD-L1 expression <1% (HR 0.94; 95% CI: 0.62–1.42). Cemiplimab plus chemotherapy also demonstrated a significant PFS benefit in the overall population, with a HR of 0.58 (95% CI: 0.47–0.72) [38]. Subgroup analyses indicated a consistent survival advantage across key baseline characteristics, including histology and PD-L1 expression levels. Furthermore, a reduction in the risk of progression or death was observed across all PD-L1 subgroups, with HRs of 0.73 (95% CI: 0.50–1.08), 0.48 (95% CI: 0.34–0.68), and 0.48 (95% CI: 0.32–0.72) for the PD-L1 <1%, 1–49%, and ≥50% populations, respectively.

Subgroup analyses from the EMPOWER-Lung3 trial demonstrate that cemiplimab plus chemotherapy provides substantial clinical benefit in patients with squamous NSCLC. In the squamous population, cemiplimab plus chemotherapy achieved a median OS of 22.3 months compared with 13.8 months for chemotherapy, corresponding to an HR of 0.61 (95% CI: 0.42–0.87). A significant improvement was also observed in PFS, with a median of 8.2 months versus 4.9 months (HR 0.56; 95% CI: 0.41–0.78). Moreover, among patients with squamous histology and PD-L1 expression ≥ 1%, ORR was significantly higher with cemiplimab plus chemotherapy at 45.3% versus 25.5% with chemotherapy (odds ratio 2.42; 95% CI: 1.14–5.11; *p* = 0.021). Collectively, these results underscore the strong therapeutic potential of cemiplimab plus chemotherapy for patients with squamous NSCLC, especially within the PD-L1 ≥ 1% subgroup.

In the safety analysis, any-grade adverse events were reported in 88.5% of patients treated with cemiplimab plus chemotherapy compared with 85.6% in the chemotherapy arm. Grade ≥ 3 adverse events occurred in 30.1% of patients in the combination arm versus 18.3% in the chemotherapy arm, and discontinuation rates due to adverse events were 4.2% and 1.3%, respectively. Although the addition of cemiplimab to chemotherapy was associated with an expected increase in adverse events, the overall safety profile was manageable and consistent with established data for other PD-1 inhibitor plus chemotherapy combinations, revealing no new or unexpected safety signals.

### 3.3. Japanese Phase I, Dose-Expansion Study

To evaluate the efficacy and safety of cemiplimab specifically in the Japanese population, a multicenter, open-label phase 1 study (NCT03233139) conducted a dose-expansion Part 2 analysis comprising two first-line treatment cohorts. The primary objectives were to assess safety, tolerability, and pharmacokinetics, while key secondary endpoints included the ORR and DOR per independent review committee, with PFS and OS assessed as exploratory endpoints [23].

In Cohort A, patients with PD-L1 expression of ≥50% received cemiplimab monotherapy (350 mg IV Q3W). Among the 50 patients with centrally confirmed PD-L1 ≥ 50%, the ORR was 60.0% (90% CI: 48.6–71.4%). Survival outcomes were robust, with a median OS of 44.5 months (95% CI: 27.0–54.4) and a 1-year survival rate of 83.7%. The median PFS was not reached (95% CI: 12.5 months–NE), with a 1-year PFS rate of 67.7%. Notably, efficacy correlated with PD-L1 expression levels; patients with PD-L1 > 90% achieved an ORR of 62.1%.

In Cohort C, patients with any level of PD-L1 expression received cemiplimab (350 mg IV Q3W) in combination with platinum-doublet chemotherapy for four cycles. In this cohort (*n* = 50), the ORR was 42.0% (90% CI: 30.5–53.5%). The median PFS was 8.1 months (95% CI: 6.0–NE), and the median OS was not reached (95% CI: 13.4–NE) at the time of data cut-off. Clinical benefit was observed regardless of PD-L1 status, including in patients with PD-L1 < 1%.

These findings in Japanese patients are consistent with the survival benefits demonstrated in the global EMPOWER-Lung 1 and EMPOWER-Lung 3 trials, supporting the use of cemiplimab as a viable therapeutic option in this population. In this Japanese phase I study, cemiplimab demonstrated a safety profile generally consistent with global trials, with no new safety signals identified. While pneumonitis occurred at 21.7% in the monotherapy cohort A (13/60; Grade ≥ 3: 5.0%, 3/60), the incidence was notably lower at 10.0% (5/50; Grade ≥ 3: 2.0%, 1/50) in the combination cohort (Cohort C). This variability suggests that the higher rate in the monotherapy cohort may be attributable to statistical fluctuations in a small sample size rather than a unique safety signal. Nevertheless, given the known susceptibility of East Asian patients to interstitial lung disease, careful monitoring is required, and further accumulation of real-world data is warranted.

## 4. Strategic Positioning in Clinical Practice

### 4.1. Cemiplimab Monotherapy: Advantages in Squamous Histology and Ultra-High PD-L1 Subgroups

Based on the results of the KEYNOTE-024 trial, pembrolizumab monotherapy has firmly established itself as the global standard for the first-line treatment of metastatic NSCLC with PD-L1 ≥ 50%, demonstrating a median OS of 26.3 months and an HR of 0.62 compared to chemotherapy [39]. However, the EMPOWER-Lung1 trial suggests that cemiplimab may possess a unique competitive edge, particularly when considering the heterogeneity of patient backgrounds; unlike KEYNOTE-024, EMPOWER-Lung1 enrolled a population with historically poorer prognostic factors, including a significantly higher proportion of squamous cell carcinoma (43.3% vs. 18.8%) and patients with clinically stable brain metastases [32] (Table 1). While subgroup analyses in KEYNOTE-024 indicated a somewhat attenuated benefit in squamous histology with an OS HR of 0.73 compared to 0.58 for non-squamous, cemiplimab demonstrated robust and consistent efficacy across histologies, notably achieving an OS HR of 0.51 in the squamous subgroup (Table 2).

A compelling opportunity for Cemiplimab to penetrate the current standard of care lies within the “ultra-high” expression population defined as PD-L1 TPS ≥ 90%, a subgroup which was pre-specified for exploratory analysis in EMPOWER-Lung1. Biologically, tumors with PD-L1 ≥ 90% are distinct from those with 50-89% expression; they are characterized by a significantly higher density of intratumoral CD8+ PD-1+ T cells and a higher frequency of BRCA2 mutations [40]. The presence of BRCA2 mutations involves defects in DNA repair genes, leading to an accumulation of somatic mutations and subsequent neoantigen production. This high TMB and neoantigen load induce a robust immune response, creating a highly immunogenic microenvironment that is also prone to the generation of ADAs due to the heightened immune activation. Given that ADA formation can neutralize therapeutic antibodies and attenuate efficacy, it is specifically within this population that cemiplimab, which is reported to exhibit lower ADA production compared to other ICIs, may yield the most substantial benefit in terms of OS and PFS. This biological rationale is corroborated by clinical data indicating superior outcomes for cemiplimab in the PD-L1 ≥ 90% population compared to available data for pembrolizumab. In EMPOWER-Lung1, the PD-L1 ≥ 90% cohort achieved a median OS of 38.8 months with a remarkable HR of 0.44. In contrast, a comparable cohort study by Ricciuti et al. reported that pembrolizumab in the PD-L1 ≥ 90% group showed a median OS of 30.4 months and an HR of 0.70 [40], with PFS comparisons also favoring cemiplimab (HR 0.51 vs. 0.69). We must compare trials carefully because study designs vary. For instance, the “ultra-high” subgroup made up about 35% of the EMPOWER-Lung 1 population. Despite this, cemiplimab shows strong potential to beat standard treatments in PD-L1 ≥ 90% tumors, especially where immunogenicity is high. While these results might not apply to all patient groups, they highlight a specific area where cemiplimab performs exceptionally well.

### 4.2. Cemiplimab Plus Chemotherapy: Clinical Value in Squamous Histology

For patients with metastatic NSCLC harboring PD-L1 expression levels of 1–49%, the combination of pembrolizumab plus chemotherapy is the established global standard of care (Table 1 and Table 3). This consensus is firmly built upon the robust long-term outcomes of the KEYNOTE-189 trial for non-squamous histology and the KEYNOTE-407 trial for squamous histology [41,42]. In KEYNOTE-189, the 5-year update demonstrated a HR for OS of 0.60, with a 5-year OS rate of 19.4%; specifically, within the PD-L1 1-49% subgroup, the HR was 0.65. Similarly, KEYNOTE-407 showed an OS HR of 0.71 and a 5-year OS rate of 18.4% for squamous patients, with the PD-L1 1-49% subgroup achieving an HR of 0.61. These data have set a high bar for any new entrant attempting to challenge this standard.

The EMPOWER-Lung3 trial, evaluating cemiplimab plus chemotherapy, introduces a unique perspective by including both squamous (42.4%) and non-squamous (57.6%) histologies within a single study design, distinguishing it from the histology-specific KEYNOTE trials (Table 1). A critical heterogeneity in patient background lies in the ethnic composition; EMPOWER-Lung3 enrolled a significantly higher proportion of Asian patients (45.1%) compared to KEYNOTE-189 (24.0%) and KEYNOTE-407 (30.6%). This demographic distinction provides substantial data for Asian populations, potentially offering an evidence-based advantage in regions where this demographic is prevalent.

Regarding efficacy, cemiplimab plus chemotherapy demonstrated a median OS of 22.3 months and an HR of 0.61 in the squamous population across all PD-L1 levels, with a 5-year OS rate of 18.0%, outcomes that are numerically comparable to the benchmark set by KEYNOTE-407 (Table 3). Although the specific Kaplan–Meier curves and detailed histology-stratified data for the PD-L1 1-49% subgroup are not fully detailed in the provided materials, the available data indicates that this regimen represents a promising therapeutic option for this specific intermediate expression group. Therefore, while pembrolizumab plus chemotherapy remains the dominant standard, cemiplimab plus chemotherapy holds potential to establish a position as a viable alternative, particularly supported by its robust performance in squamous cell carcinoma and its unique dataset enriched with Asian patients.

## 5. Conclusions and Future Perspectives

Cemiplimab represents a sophisticated refinement in the class of anti-PD-1 therapies. The engineering of the S228P mutation ensures molecular stability and minimizes immunogenicity—evidenced by ADA rates significantly lower than those of atezolizumab and nivolumab—while its unique reliance on the N58-glycan for binding allows for high-affinity receptor occupancy that distinctively positions it against pembrolizumab.

These molecular features have translated into distinctive clinical benefits demonstrated in the EMPOWER-Lung program, which identified two distinct “therapeutic niches” where cemiplimab excels. Firstly, in the PD-L1 ≥ 90% “ultra-high” population, cemiplimab offers a median survival of nearly 39 months with monotherapy alone, providing the strongest evidence to date for this subgroup. Furthermore, in squamous cell carcinoma, cemiplimab provides a highly effective combination option with a median survival exceeding 22 months, thereby challenging historical norms for this difficult-to-treat histology. However, it is important to note that the efficacy data for the PD-L1 ≥ 90% and squamous cell carcinoma populations are derived from subgroup analyses. Therefore, these findings warrant cautious interpretation, and the further accumulation of real-world data is essential to validate these results.

In the setting of Stage IV NSCLC, while randomized controlled trials have demonstrated robust efficacy, the continued accumulation of real-world data remains essential to validate these benefits across diverse patient populations in routine clinical practice. Looking beyond metastatic disease, the potential of cemiplimab is expanding into earlier stages. Ongoing studies are currently assessing the drug for adjuvant and neoadjuvant use in NSCLC. Specific examples include trials NCT06931717, NCT06449313, and NCT06465329. Given the positive results already seen with cemiplimab in adjuvant/neoadjuvant cutaneous squamous cell carcinoma [32,43], there is a strong biological rationale to anticipate similar success in lung cancer.

In conclusion, the approval of cemiplimab in Japan offers clinicians a powerful new option that allows for a more personalized approach to lung cancer treatment. Cemiplimab fills a specific gap for treating patients with squamous histology and ultra-high PD-L1 levels. Its success is likely due to its stable structure and low immunogenicity.

## Figures and Tables

**Table 1:** Patient Characteristics Summary.

Characteristic	EMPOWER-Lung 1	EMPOWER-Lung 3	KEYNOTE-024	KEYNOTE-189	KEYNOTE-407
Study Design					
Regimen	Cemiplimab	Cemiplimab + Chemotherapy	Pembrolizumab	Pembrolizumab + Chemotherapy	Pembrolizumab + Chemotherapy
Target Population	PD-L1 ≥ 50%	All Comers	PD-L1 ≥ 50%	Non-Squamous	Squamous
Demographics					
Median Age	63 years	64 years	64.5 years	65 years	65 years
Gender (Male)	87.7%	70.0%	59.7%	65.8%	80.5%
ECOG PS 1	72.9%	63.8%	64.3%	60.2%	70.3%
Histology					
Squamous	43.3%	42.4%	18.8%	0.0%	100.0%
Non-Squamous	56.7%	57.6%	81.2%	100.0%	0.0%
PD-L1 Status					
<1%	-	32.0%	-	32.9%	35.1%
1–49%	-	36.0%	-	36.0%	38.8%
≥50%	100.0%	32.0%	100.0%	31.2%	26.1%
≥90% (Ultra-high)	35.0%	Not Reported	Not Reported	Not Reported	Not Reported

**Table 2:** Efficacy Data for Monotherapy.

Trial Name	Agent	Target Population	Histology	Median OS (95% CI)	OS HR (95% CI)	Median PFS (95% CI)	PFS HR (95% CI)
EMPOWER-Lung 1	Cemiplimab	Overall (PD-L1 ≥ 50%)	All	26.1 months (22.1–31.9)	0.59 (0.48–0.72)	8.1 months (6.2–8.8)	0.50 (0.41–0.61)
		PD-L1 ≥ 90% (Ultra-high)	All	38.8 months (22.9–NE)	0.44 (0.30–0.65)	14.7 months (10.2–21.1)	0.32 (0.22–0.46)
		PD-L1 ≥ 50%	Squamous	22.7 months (17.3–31.5)	0.51 (0.38–0.69)	8.3 months (5.6–9.4)	0.44 (0.32–0.60)
		PD-L1 ≥ 50%	Non-Sq	28.7 months (22.9–44.3)	0.66 (0.50–0.88)	6.5 months (4.4–12.4)	0.55 (0.42–0.72)
Japanese Phase 1	Cemiplimab	Cohort A (PD-L1 ≥ 50%)	All	44.5 months (27.0–54.4)	-	NR (12.5–NE)	-
KEYNOTE-024	Pembrolizumab	Overall (PD-L1 ≥ 50%)	All	26.3 months (18.3–40.4)	0.62 (0.48–0.81)	7.7 months (6.1–10.2)	0.50 (0.39–0.65)
		PD-L1 ≥ 50%	Squamous	-	0.61 (0.30–1.24)	-	0.37 (0.19–0.73)
		PD-L1 ≥ 50%	Non-Sq	-	0.63 (0.47–0.84)	-	0.54 (0.41–0.71)

Abbreviations: OS, Overall Survival; PFS, Progression-Free Survival; NR, Not Reached; NE, Not Evaluable; HR, Hazard Ratio (vs. Chemotherapy), CI: Confidence Interval. Note: Figures for EMPOWER-Lung 1 are based on the 5-year update. The analysis focuses on the modified intention-to-treat population with confirmed PD-L1 levels of 50% or higher.

**Table 3:** Efficacy Data for Combination Therapy.

Trial Name/ Agent	Target Population (Subgroup)	Histology	Median OS (95% CI)	OS HR (95% CI)	Median PFS (95% CI)	PFS HR (95% CI)
EMPOWER-Lung 3/ Cemiplimab + Chemo	Overall	All	21.1 months (15.9–23.9)	0.66 (0.53–0.83)	8.2 months (6.4–9.3)	0.58 (0.47–0.72)
	Squamous (PD-L1 any)	Squamous	22.3 months (15.7–27.2)	0.61 (0.42–0.87)	8.2 months (6.3–10.7)	0.56 (0.41–0.78)
	Non-Squamous (PD-L1 any)	Non-Squamous	19.4 months (14.0–23.5)	0.64 (0.47–0.88)	-	0.61 (0.45–0.81)
	PD-L1 1–49%	All	-	0.50 (0.34–0.74)	-	0.48 (0.34–0.68)
Japanese Phase 1/ Cemiplimab + Chemo	Cohort C (Overall)	All	NR (13.4–NE)	-	8.1 months (6.0–NE)	-
	PD-L1 1–49%	All	-	-	-	-
KEYNOTE-189/ Pembrolizumab + Chemo	Overall	Non-Squamous	22.0 months (19.5–24.5)	0.60 (0.50–0.72)	9.0 months (8.1–10.4)	0.50 (0.42–0.60)
	PD-L1 1–49%	Non-Squamous	21.8 months (17.7–25.6)	0.65 (0.46–0.90)	9.4 months (6.1–12.8)	0.57 (0.41–0.80)
KEYNOTE-407/ Pembrolizumab + Chemo	Overall	Squamous	17.2 months (14.4–19.7)	0.71 (0.59–0.85)	6.4 months (6.2–8.3)	0.62 (0.52–0.74)
	PD-L1 1–49%	Squamous	19.3 months (12.2–25.2)	0.61 (0.45–0.83)	6.5 months (4.4–12.4)	0.57 (0.42–0.78)

Abbreviations: OS, Overall Survival; PFS, Progression-Free Survival; NR, Not Reached; NE, Not Evaluable; HR, Hazard Ratio, CI: Confidence Interval.

## Data Availability

No new data were created or analyzed in this study. Data sharing is not applicable to this article.
